# The effect of infantile anti-colic massage and kangaroo care on postpartum anxiety, postpartum depression and maternal function of mothers*

**DOI:** 10.1590/1980-220X-REEUSP-2025-0146en

**Published:** 2026-01-23

**Authors:** Gülçah Sunay Ertem, Nurcan Özyazıcıoğlu

**Affiliations:** 1 Balıkesir State Hospital, Balıkesir, Türkiye.; 2 Bursa Uludağ University, Faculty of Health Sciences, Department of Pediatric Nursing, Bursa, Türkiye.

**Keywords:** Massage, Kangaroo-Mother Care Method, Anxiety, Depression, Postpartum, Massagem, Método Canguru, Ansiedade, Depressão Pós-Parto

## Abstract

**Objective:**

This study aimed to determine the effects of postpartum colic massage and kangaroo care on anxiety, family and maternal productivity in mothers.

**Method:**

This study is a randomized controlled trial. The study was conducted with mothers of infants diagnosed with infantile colic. The study groups were randomly divided into three groups, namely massage, kangaroo care and control group, by stratified randomization. Study data were collected using the “Mother-Baby Information Form”, “The Barkin Index of Maternal Functioning (BIMF)”, “Postpartum Specific Anxiety Scale” and “Beck's Depression Inventory (BDI)”. Data collection was completed between March 2022 and February 2023. The study was completed with a total of 90 mothers.

**Results:**

The findings indicated that maternal functioning showed the greatest improvement in the massage group (12.31 ± 5.93, p < 0.001). The decrease in postpartum anxiety and depression scores was smaller in the control group compared with the massage and kangaroo care groups (5.29 ± 9.51, 1.39 ± 1.28). A significant negative correlation was observed between infantile colic and maternal functioning (rs = -0.69, p < 0.001), whereas significant positive correlations were found between infantile colic and postpartum anxiety and depression (rs = 0.51, rs = 0.58, p < 0.001).

**Conclusion:**

Colic massage and kangaroo care should be integrated into routine prenatal and postpartum education and should be applied by nurses in clinical care.

## RESUMO

**Objetivo:**

Este estudo teve como objetivo determinar os efeitos da massagem pós-parto para cólicas e do método canguru na ansiedade, na família e na produtividade materna das mães.

**Método:**

Este estudo é um ensaio clínico randomizado. O estudo foi realizado com mães de bebés diagnosticados com cólica infantil. Os grupos do estudo foram divididos aleatoriamente em três grupos, nomeadamente massagem, método canguru e grupo de controlo, por randomização estratificada. Os dados do estudo foram recolhidos utilizando o «Formulário de Informação Mãe-Bebé», o «Índice Barkin de Funcionamento Materno (BIMF)», a «Escala de Ansiedade Pós-parto Específica» e o «Inventário de Depressão de Beck (BDI)». A recolha de dados foi concluída entre março de 2022 e fevereiro de 2023. O estudo foi concluído com um total de 90 mães.

**Resultados:**

Os resultados indicaram que o funcionamento materno apresentou a maior melhoria no grupo da massagem (12,31 ± 5,93, p < 0,001). A diminuição nos escores de ansiedade e depressão pós-parto foi menor no grupo de controlo em comparação com os grupos da massagem e do método canguru (5,29 ± 9,51, 1,39 ± 1,28). Observou-se uma correlação negativa significativa entre a cólica infantil e o funcionamento materno (rs = –0,69, p < 0,001), enquanto foram encontradas correlações positivas significativas entre a cólica infantil e a ansiedade e depressão pós-parto (rs = 0,51, rs = 0,58, p < 0,001).

**Conclusão:**

A massagem para cólicas e o método canguru devem ser integrados na educação pré-natal e pós-parto de rotina e devem ser aplicados por enfermeiras no atendimento clínico.

## INTRODUCTION

Infantile colic is defined as excessive crying lasting more than three hours per day, at least three days a week, and persisting for longer than three weeks within the first three months of life. This condition was first described by Wessel and colleagues in 1954^(1)^. Infantile colic is widely recognized as a highly distressing experience for parents and is particularly associated with psychological and familial challenges among first-time mothers^(2)^.

Studies have shown that prolonged and intense crying episodes in colicky infants lead to feelings of inadequacy and helplessness in parents, contribute to postpartum depression, and increase the need for social support^(3,4)^. Mothers of colicky infants have been reported to experience higher levels of depression, insecure attachment, and postpartum anxiety compared to mothers of non-colicky infants^(5,6)^. Maternal postpartum stress and depression cause mothers to question their caregiving competence, thereby making it difficult to provide safe and adequate care for their infants^(6)^. Furthermore, increased parental stress has been reported to exacerbate the symptoms of infantile colic^(7)^. Another study identified a significant association between high maternal stress levels and greater severity of colic symptoms^(8)^. Moreover, research has shown that maternal psychological problems during the prenatal period contribute to crying and irritability behaviors in infants^(9,10)^.

A systematic review examining early parenting difficulties emphasized the interdependence of child and parental well-being: interventions that improve parents also have positive effects on children, whereas interventions that benefit children support the psychosocial adjustment of parents^(11)^. This finding underscores that parent–child interaction is a bidirectional process and represents a reciprocal cycle of improvement within family dynamics. In the postpartum period, parental support programs, psychoeducational interventions, infant-centered care approaches, and family-centered health services have been shown to reduce maternal stress levels and improve emotional well-being^(11,12,13)^. Infant massage and kangaroo care have been found to decrease maternal stress and anxiety levels and to reduce physiological stress markers^(14)^. Studies have also shown that these practices strengthen mother–infant bonding, support oxytocin release, facilitate early acceptance of the maternal role, and enhance maternal self-efficacy and sensitivity in caregiving^(15,16,17)^.

Despite these promising findings, nursing interventions specifically designed to support maternal well-being and role adaptation among mothers of colicky infants remain limited. Moreover, most existing studies have predominantly focused on interventions described as “infant massage,” leaving gaps in understanding the comparative effectiveness of different approaches.

In conclusion, new high-quality studies are needed to develop coping strategies for colic management, to support maternal well-being, to determine which interventions are more effective, and to address gaps in the literature. In line with this, the present study was conducted to determine the effects of two *interventions “*Colic Massage” and *“*Kangaroo Care” on postpartum depression, postpartum anxiety, and maternal functioning.

## METHOD

### Study Design

This study is a pretest-posttest, experimental, randomized controlled trial conducted to determine the effects of anti-colic massage and kangaroo care on maternal function, postpartum anxiety, and postpartum depression levels in mothers of infants diagnosed with colic according to the Rome IV criteria.

### Place and Time of the Study

Data for the study were collected at the outpatient clinic of the Department of Pediatrics at Atatürk City Hospital on two randomly selected days per week. Data were collected from mothers of infants brought to the outpatient clinic for examination and diagnosed with infantile colic for the first time by a pediatrician. Data collection was completed between March 2022 and February 2023.

### Research Population and Sample

Infants with newly diagnosed infantile colic and their mothers who met the inclusion criteria were included in the study between March 2022 and April 2023.

### Sample Size

The sample size of the study was determined using G*Power 3.1.9.7. The effect size was calculated for both infants and mothers using the repeated measures ANOVA approach based on studies in the literature. Since the effect size of studies on mothers was found to be lower, calculations based on mothers were used to ensure comprehensiveness. Accordingly, with an effect size of f = 0.340, a power of 0.80 (1-β error probability), and α = 0.05 (Type I error probability), the minimum required sample size for a design with 3 groups and 2 repeated measurements was calculated to be 28 participants per group, totaling 84 participants^(5,18,19)^. Since there might be losses during the study, the sample size was increased by 15% to 96. The group sizes were determined to have 32 mothers in each group (massage, kangaroo care, and control group). A total of 102 infants and mothers were reached during the study process.

**Figure 1:**
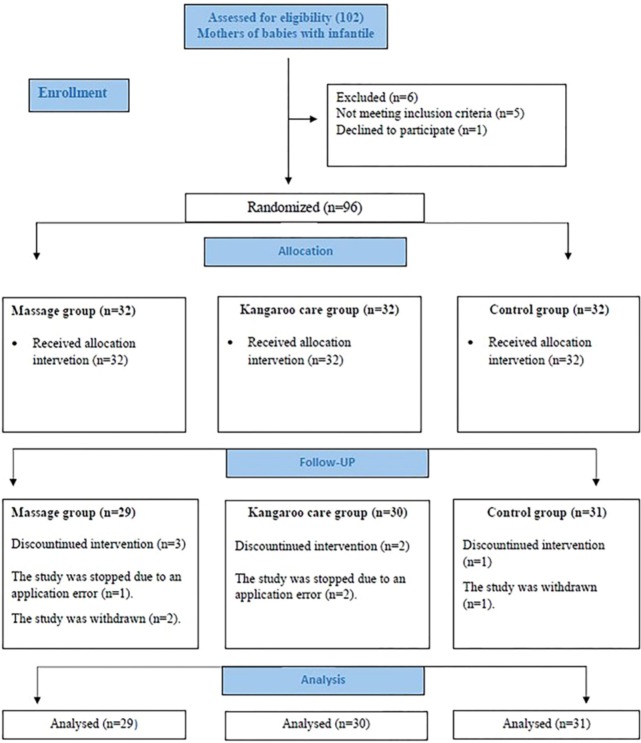
CONSORT flow diagram.

Six mother-infant pairs were not included in the study as they did not meet the exclusion criteria. During the study, six mothers dropped out for various reasons. The study was completed with 90 mothers (Figure 1).

### Inclusion Criteria

The study included full-term male and female infants weighing between 2500 and 4500 grams, diagnosed with infantile colic according to Rome IV criteria, and their mothers. Mothers who volunteered to participate in the study, could read and write Turkish, and could use a smartphone were also included.

### Exclusion Criteria

Infants with congenital anomalies, lactose intolerance, acute or chronic illnesses, skin problems, vomiting or diarrhea, a birth weight of less than 2500 grams, receiving treatment for colic, or developing any illness during the study period were excluded from the study. Additionally, mothers who failed to implement at least one of the routine interventions, implemented the intervention incorrectly, or were unwilling to continue the study were excluded from the study and considered lost.

### Randomization and Allocation

Studies in the literature indicate that age (weeks) and sex are prognostic factors affecting infant colic symptoms^(12,18,19)^. Therefore, stratified randomization was used to assign infants and their mothers to intervention and control groups. To control for the effects of age and sex on colic pain and to increase the reliability of the results, infants were stratified by age (3–8 weeks) and sex (male-female). Randomization of infants was performed by randomly entering all numbers from 1 to 96 via https://www.randomizer.org. The first number drawn was assigned to the massage group, the second to the kangaroo care group, and the third to the control group.

### Measurements

Study data were collected using the “Mother-Baby Information Form”, “The Barkin Index of Maternal Functioning (BIMF)”, “Postpartum Specific Anxiety Scale” and “Beck’s Depression Inventory (BDI)”.

***Mother-Baby Information Form:*** This form, developed by the researcher as a result of literature review, was finalized in collaboration with a faculty member in the field of pediatric nursing^(2,3,4,5,9,10)^. “The form included variables such as mothers’ age, education level, employment and income status, number of children, family type, tobacco and alcohol use, cow’s milk use, home heating method, presence of allergies, and whether they received support with baby care. It also included information on hospital visits during colic attacks”^(20)^. “Additionally, the form included variables related to the infants, such as age, sex, mode of delivery, anthropometric measurements at birth and at the current period, birth order, feeding method, bottle and pacifier use, daily feeding frequency, daily defecation frequency, and the onset time and characteristics of crying episodes”^(20)^.

***The Barkin Index of Maternal Functioning (BIMF):*** This scale was developed in 2010 to determine the functional status of the mother after birth^(18)^. The original Cronbach alpha coefficient of the scale is 0.87^(18)^. As a result of group-focused interviews with 31 women, a scale consisting of 20 questions was prepared, validity and reliability studies were conducted and published^(18)^. The validity and reliability study of the Turkish form of the BAFÖ was completed by Aydın and Kukulu in 2018^(19)^. Since items 15, 16, 18 and 20 of the form were not deemed appropriate, a 7-point Likert-type, 16-question, 5-factor Turkish scale was created and the reliability coefficient was determined to be 0.73. The Cronbach alpha coefficients of the sub-dimensions of the scale are as follows: self-care 0.66, maternal psychology 0.71, baby care 0.62, social support 0.69, and the dimension of adaptation to motherhood was calculated as 0.50. The responses on the scale are numbered between 0 and 6. 0 is expressed as “I completely disagree”, 1 is “I disagree”, 2 is “I somewhat disagree”, 3 is “I am undecided”, 4 is “I somewhat agree”, 5 is “I agree” and 6 is “I completely agree”. The lowest score that can be obtained in scoring the scale is 0, and the highest score is 96. It is accepted that the functional status increases as the scores increase. BAFÖ is applied to mothers with babies younger than 18 months^(19)^. The Cronbach Alpha coefficient value of the BAFÖ was found to be 0.87^(19)^.

***Beck’s Depression Inventory (BDI):*** Beck depression scale was developed in 1961 to measure the behavioral findings of depression in adolescent and adults^(21)^. The original Cronbach alpha coefficient of the scale is 0.86^(21)^. The validity and reliability of the scale was made by Hisli in 1989 and the Cronbach alpha value was found to be 0.80^(22)^. The scale consists of 21 items related to depressive symptoms such as pessimism, feeling of failure, dissatisfaction, feelings of guilt, restlessness, fatigue, decreased appetite, indecisiveness, sleep disturbance, and social withdrawal. The total score that can be obtained from the scale varies between 0 and 63. In the original version of the scale, borderline clinical depression was determined as 17–20 points. In the validity and reliability of the scale in Turkish society, a score of 17 and above was accepted as the level of “clinically significant depression”. Validity and reliability studies of the BDI in Turkish the severity of depression is interpreted as BDI scoring as 0-9 = Minimal, 10-16 = Mild, 17-29 = Moderate, 30-63 = severe, depression^(22)^.

***Postpartum Specific Anxiety Scale:*** The Postpartum Specific Anxiety Scale is a 51-item scale developed by Fallon and colleagues to assess anxiety^(23)^. The original Cronbach alpha coefficient of the scale is 0.95, and the subscales range between 0.80–0.91^(23)^. In the Turkish validity and reliability study of the Postpartum Specific Anxiety Scale (PSAS), the Cronbach’s alpha coefficient was found to be 0.94^(24)^. Responses to the items are rated on a 4-point Likert scale ranging from 1 to 4 points (1 = never, 2 = sometimes, 3 = usually, 4 = always). Total postpartum anxiety level score; below 73 points is considered low risk, between 74 and 100 points is considered medium risk, and 101 points and above is considered high risk. The lowest possible score is 51, and the highest score is 204. The scale has four sub-dimensions: Items 1–15 assess maternal adequacy and attachment concerns, Items 16–26 assess infant safety and well-being concerns, Items 27–33 assess practical infant care concerns, and Items 34–51 assess psychosocial adjustment to motherhood^(24)^.

## EDUCATIONAL MATERIALS USED IN THE STUDY

### Educational Massage Application Checklist

The “Educational Massage Application Checklist” was created by the researcher based on the literature and the opinion of a faculty member of the Department of Child Health and Disease Nursing. This three-part educational material provided information on the benefits of baby massage, preparation before massage, duration of massage, and situations in which massage should not be applied^(25,26,27)^. It was also supported by pictures showing the detailed stages of colic massage. In addition, a chart consisting of yes-no questions was added so that the mother could follow the massage order while applying the massage.

### Educational Kangaroo Care Application Checklist

The “Educational Kangaroo Care Application Checklist” was developed by the researcher based on the literature and the expert opinion of a faculty member from the Department of Child Health and Disease Nursing^(28)^. The educational material consisted of three sections and included information on the benefits of kangaroo care, who could apply it, when and for how long it should be applied, the proper positioning of the baby, and the appropriate environment for kangaroo care. It also contained images illustrating the stages of kangaroo care application. Additionally, the material included a chart with yes/no questions to help mothers follow the correct order and rules of kangaroo care practice.

### Model Baby

The baby training manikin was used for demonstrating colic massage and kangaroo care.

### Massage Oil

Hypoallergenic baby massage oil was used in the massage classes to provide lubrication for the babies.

### Thermometer

The thermometer was used to ensure the environment was at an appropriate temperature during massage and kangaroo care.

## DATA COLLECTION AND INTERVENTIONS

Infants diagnosed with infantile colic by a pediatrician according to Rome IV criteria and their mothers participated in the study. Rome IV diagnostic criteria are as follows:

1. Symptoms must cease within the first five months of onset.
2. Recurrent, prolonged crying spells must occur without an obvious cause and cannot be stopped by caregivers.
3. The baby must not have any signs of illness or growth or developmental delays^(29)^.

### Control Group Intervention

Mothers whose babies were diagnosed with infantile colic were invited to participate in the study, and the volunteer mothers filled out the “Informed Consent Form” and “Mother-Baby Information Form”. Then, the mothers were administered the “Barkin Maternal Function Scale (BIMF), Postpartum Specific Anxiety Scale”, “Beck Depression Inventory” and the “Infantile Colic Scale”. Three weeks after the first tests, the same tests were administered to the mothers in the control group.

### Massage Group and Kangaroo Care Group Interventions

Data were collected from volunteer mothers of infants diagnosed with infantile colic who met the inclusion criteria of the study. The mothers were informed about the study using the “Informed Consent Form,” and the data were gathered through face-to-face interviews. Before the interventions, mothers in both groups completed the “Barkin Maternal Functioning Scale (BIMF),” the “Beck Depression Inventory”, “Postpartum Specific Anxiety Scale” and the “Infantile Colic Scale”. After the pre-tests were completed and before the intervention phase began, two home visit appointments were scheduled with the mothers in the intervention group, one week apart. Two home visits were carried out in total. During the second visit, mothers in the massage group were taught colic massage, while mothers in the kangaroo care group were taught kangaroo care. In addition, the mothers in the intervention group continued to provide routine care for their babies throughout the period in which they performed the colic massage. The duration of the intervention was determined based on the literature and the implementation periods used in studies similar to ours^(30,31,32,33)^.

### The Intervention Applied to The Massage Group

The researcher nurse provided comprehensive, face-to-face, individual information to the mothers in the massage group about colic massage during home visits. The research nurse then demonstrated how to perform colic massage on a baby dummy. The previously demonstrated colic massage technique was then performed by the mothers on the baby dummy under the supervision of the research nurse. The mothers repeated the massage until they were able to perform it correctly. This training and demonstration by the researcher was given to the mothers in a single session before the study began (the researcher held a holistic baby massage certificate). After the training and instruction, the research nurse also provided the mothers with the “Colic Massage Education Checklist.” Following this information and demonstration, the mothers began to independently perform colic massage on their babies at home. For three weeks, the mothers performed a two-stage colic massage on their babies according to the “Colic Massage Education Checklist.” For three weeks, they performed colic massage for 15–20 minutes in the morning and evening when their babies were calm. Additionally, when babies began to show signs of discomfort, mothers performed the second phase of colic massage for 15 minutes^(34)^. The colic massage (CM) consisted of two parts: the “I Love You” massage, which was applied when the infant was calm, and another massage technique used when the infant was restless. These techniques included:

1. When the infant was calm, the massage was applied to the abdomen while the infant lay in a supine position. The letters “I,” inverted “L,” and inverted “U” were gently traced on the infant’s abdomen in sequence. While maintaining the same position, the infant’s knees were bent. This massage sequence was repeated several times.
2. When the infant was restless, the massage was performed with the infant held facing forward in the mother’s lap. The mother passed her left arm under the infant’s armpit to support the chest and, using her right hand, gently traced an inverted “U” shape on the infant’s abdomen in a clockwise direction. She then gently stroked the infant’s abdomen from top to bottom with her right hand. Finally, while the infant remained in the mother’s lap, the mother bent the infant’s knees and gently bounced and rocked the infant side-to-side^(25,26,27,28)^.

### The Intervention Applied to the Kangaroo Care Group

During home visits, the research nurse provided comprehensive, face-to-face, and individualized information about kangaroo care to the mothers in the massage group. The research nurse then demonstrated how to perform kangaroo care on a dummy baby. The mothers, under the supervision of the research nurse, performed the kangaroo care procedure on the dummy baby. The mothers repeated the kangaroo care procedure until they were able to perform it correctly. This training and demonstration, provided by the researcher, was given to the mothers in a single session before the study began. Following the training and demonstration, the research nurse gave the mothers the “Kangaroo Care Education Checklist.” Following this home visit by the research nurse, the mothers began independently performing kangaroo care on their babies at home. They applied kangaroo care for 15–20 minutes in the morning and evening when the baby was calm, and for an additional 15 minutes when the baby began to show signs of discomfort^(17,33)^.

After the interventions ended, the mothers in both groups filled out the “Barkin Maternal Functioning Scale (BIMF),” the “Beck Depression Inventory” “Postpartum Specific Anxiety Scale.” and the “Infantile Colic Scale”. Throughout the intervention phase, mothers received support and guidance via video calls as needed. When video calls were not feasible, participants were asked to submit video recordings of their practice sessions. (The research nurse had the application done as an example in the videos requested from the mothers and in the video interviews she had with the mothers. The research nurse was not involved in the application that the mothers had to do within the scope of the research. The mother did the morning and evening applications specified in the application rules alone. Mothers were contacted via WhatsApp to remind them about the interventions. This ensured that the interventions were carried out within the specified time. The study lasted a total of four weeks, with the intervention phase occupying the first three weeks.

### Data Analysis

Data entry into the statistical program was performed by assigning codes to the groups. The intervention and control groups were coded as 1, 2, and 3. The person conducting the data analysis was blinded to the group assignments. The Shapiro-Wilk test was used to assess the normality of continuous variables. Continuous variables were presented as mean, standard deviation, or median (minimum: maximum) values. Categorical variables were expressed as n (%). If the data were normally distributed or if there were two or more groups, the ANOVA test was used; otherwise, the Kruskal-Wallis test was applied. If overall significance was found in the subgroup advanced analysis, ANOVA was followed by Bonferroni test, Kruskal-Wallis test was followed by Dunn-Bonferroni test. (Our study’s advanced statistical analyses are presented in Table 4). In the comparison of pre-test and post-test results, if normality was observed, the paired sample t-test was used; otherwise, the Wilcoxon signed-rank test was applied. Additionally, the Chi-square test and Fisher Freeman Halton tests were used for the comparison of categorical variables between groups. Statistical analyses were performed using SPSS software (IBM Corp. Released 2012. IBM SPSS Statistics for Windows, Version 25.0. Armonk, NY: IBM Corp.), and the Type I error level was set at 5%. Addinationaly the core longitudinal comparisons in the manuscript were based on pre-versus post-intervention change scores, evaluated with appropriate within-group and between-group tests chosen according to the data’s distributional properties (parametric when assumptions held, and nonparametric otherwise). We did not perform a classical repeated-measures ANOVA to estimate a Group × Time interaction. We did not plan to introduce post hoc mixed-effects models or other “advanced” interaction tests, because key statistical assumptions required for those models were violated in several primary outcomes. Specifically, different distributions exhibited substantial deviations from normality, variance heterogeneity across groups was present, and for some variables, the data structure (e.g., skewness and presence of outliers in outcomes like crying duration) would render interaction estimates from parametric factorial models unreliable or misleading. Forcing a Group × Time interaction test under these conditions—without reliable correction or a substantially larger sample to stabilize estimates—would risk generating spurious findings (inflated Type I error from sphericity or homoscedasticity violations), biased or unstable effect estimates, and overconfident inference (narrowed confidence intervals that do not reflect true uncertainty). Likewise, introducing complex models at this stage would border on overfitting and could obscure rather than clarify the intervention’s practical signal. Therefore, we judged that the originally reported approach, centered on change scores with appropriate test selection, provides the most transparent and statistically defensible summary of differential change over time between groups, given the data at hand.

### Ethical Issues

This study was conducted in accordance with the Declaration of Helsinki. Ethical approval for the study was received from the Non-Interventional Research Ethics Committee of the Health Sciences University of Bandırma On Yedi Eylül (approval numbered 72 dated 17.12.2021). Institutional permission to conduct the study at Atatürk City Hospital was given by the Balıkesir Provincial Health Directorate (Commission approval dated 07.03.2022). All ethical guidelines for human research were followed. Written consent was obtained from the mothers via the “Informed Consent Form” before data collection. Participants were informed that they could withdraw from the study at any time.

## RESULTS

The data collected in line with the objectives of the pre-test-post-test experimental design study to reveal the effect of anti-colic massage and kangaroo care on colic babies were analyzed statistically. The socio-demographic characteristics of the mothers were compared between the groups. Data revealed that there was no difference between the groups in terms of mothers’ age, education, pregnancy period, number of children, family type, mother’s receiving help and planned pregnancy (Table 1).

**Table 1:** Comparison of mothers’ sociodemographic characteristics between groups - Balıkesir, Marmara Region, Turkey, 2023.

Characteristics	Massage group		Kangaroo care group		Control group		p value
	n	%	n	%	n	%	
**Mother’s Education Level**							
Primary Education	4	13.80	3	10	1	3.20	0.689^b^
High School	9	31	11	36.70	11	35.50	
Undergraduate and Graduate	16	55.20	16	53.30	19	61.30	
**Pregnancy period**							
Good (Healty)	20	69	17	56.70	13	41.90	0.108^a^
Bad (With health problems)	9	31	13	43.30	18	58.10	
**Family type**							
Nuclear Family	20	69	20	66.70	21	67.70	0,919^b^
Extended Family	3	10.30	4	13.30	2	6.50	
Temporary Extended Family	6	20.70	6	20	8	25.80	
**Mother’s Support Status**							
Yes	22	75.90	24	80	23	74.20	0.859^a^
No	7	24.10	6	20	8	25.80	
**Planned Pregnancy**							
Yes	17	58.60	17	56.70	16	51.60	0.852^a^
No	12	41.40	13	43,30	15	48.40	
**Number of children of the mother**	1.79 ± 0.82		1.70 ± 0.65		1.77 ± 0.72		0.937^c^
**Mother’s age**	28.79 ± 4.99		28.33 ± 4.59		28.97 ± 5.08		0.875^c^

Data are expressed as mean ± st deviation and n%.

a: Chi-square test,

b: Fisher-Freeman-Halton test,

c: Kruskal Wallis test.

The mothers’ intragroup scores in the Barkin Index of Maternal Functioning (BIMF), Postpartum Specific Anxiety Scale and Beck’s Depression Inventory (BDI) were compared (Table 2). The post-test Barkin Index of Maternal Functioning scores of the mothers in the massage, kangaroo care and control groups were found to be higher compared to their pre-test scores (p < 0.001). The post-test Postpartum Specific Anxiety Scale scores of the mothers in the massage, kangaroo care and control groups were found to be lower compared to their pre-test scores (p < 0.001, Table 2). The post-test Beck’s Depression Inventory scores of the mothers in the massage, kangaroo care and control groups were found to be lower compared to their pre-test scores (p < 0.001, Table 2).

**Table 2:** Intragroup comparison of mothers' barkin index of maternal functioning, postpartum specific anxiety and beck depression scale scores - Balıkesir, Marmara Region, Turkey, 2023.

	Massage group	Kangaroo care group	Control group
The Barkin Index of Maternal Functioning (BIMF) Pre-test score	67.62 ± 11.25	74.83 ± 6.18	65.97 ± 6.78
The Barkin Index of Maternal Functioning (BIMF) Post-test score	79.93 ± 8.24	82.63 ± 7.42	68.48 ± 7.50
p value	**< 0.001^e^**	**< 0.001^e^**	**< 0.001^e^**
Postpartum Specific Anxiety Pre-test Score	100.69 ± 20.96	95.13 ± 15.87	109.03 ± 14.55
Postpartum Specific Anxiety Post-test Score	79.48 ± 15.89	82.47 ± 14.88	103.74 ± 10.84
p value	**< 0.001^d^**	**< 0.001^e^**	**0.004^e^**
Beck Depression Inventory Pre-test Score	18.14 ± 8.80	12.57 ± 4.97	13.48 ± 3.41
Beck Depression Inventory Post-Test Score	10.41 ± 5.17	9.33 ± 4.47	12.09 ± 3.18
p value	**< 0.001^e^**	**< 0.001^d^**	**< 0.001^d^**

Data are expressed as mean ± st deviation.

d: Wilcoxon Signed Rank test,

e: Dependent Sample t test.

The difference between the pre-test and post-test scores of mothers in the Barkin Index of Maternal Functioning, Postpartum Specific Anxiety Scale, and Beck’s Depression Inventory was calculated (Table 3). The increase in the Barkin Index of Maternal Functioning scores of mothers in the control group was found to be lower compared to those in the massage and kangaroo care groups (p < 0.001 and p < 0.001, Table 3). The decrease in the Postpartum Specific Anxiety Scale scores of mothers in the control group was found to be lower compared to those in the massage and kangaroo care groups (p < 0.001 and p < 0.002, Table 3). No difference was observed between the massage and kangaroo care groups in terms of maternal functioning and postpartum anxiety post-test scores (p = 0.090 and p = 0.104, Table 3). The decrease in the Beck’s Depression Inventory scores of mothers in the massage group was found to be higher compared to those in the control and kangaroo care groups (p = 0.001 and p < 0.001, Table 3). The decrease in the Beck’s Depression Inventory scores of mothers in the kangaroo care group was found to be higher compared to the control group (p = 0.013, Table 3).

**Table 3:** Comparison of mothers' intergroup difference scores on barkin maternal functioning index, postpartum specific anxiety and beck depression scales - Balıkesir, Marmara Region, Turkey, 2023.

					Subgroup analyses		
	Massage group	Kangaroo care group	Control group	p value	Massage and Kangaroo care group p value	Massage and control group p value	Kangaroo care and control group p value
Barkin Index of Maternal Functioning (BIMF) Difference Score	12.31 ± 5.93	7.80 ± 4.70	2.51 ± 2.60	**< 0.001^c^**	0.090	**< 0.001**	**< 0.001**
Postpartum Specific Anxiety Difference Score	21.21 ± 12.97	12.67 ± 8.8	5.29 ± 9.51	**< 0.001^c^**	0.104	**< 0.001**	**0.002**
Beck Depression Scale Difference Score	-7.72 ± 4.59	3.23 ± 2.88	1.39 ± 1.28	**< 0.001^c^**	**0.001**	**< 0.001**	**0.013**

Data are expressed as mean ± st,

c: Kruskal Wallis Test,

f: ANOVA Test.

The relationship between infants’ infantile colic symptoms and mothers’ maternal functioning, postpartum anxiety, and depression status was calculated (Table 4). A significant inverse correlation was found between the difference score of the Infantile Colic Scale and the difference score of the Barkin Maternal Functioning Scale (rs = -0.69, p < 0.001). A significant positive correlation was found between the difference score of the Infantile Colic Scale and the difference score of Postpartum-Specific Anxiety (rs = 0.51, p < 0.001). A significant positive correlation was found between the difference score of the Infantile Colic Scale and the difference score of the Beck Depression Inventory (rs = 0.58, p < 0.001).

**Table 4:** Correlation of infantile colic scale difference score with barkin maternal function, postpartum specific anxiety and beck depression inventory difference scores - Balıkesir, Marmara Region, Turkey, 2023.

	Infantile colic scale difference score
Barkin Index of Maternal Functioning (BIMF) Difference Score	r_s_ = -0,69 p = **< 0,001**
Postpartum Specific Anxiety Difference Score	r_s_ = 0,51 p = **< 0,001**
Beck Depression Scale Difference Score	r_s_ = 0,58 p = **< 0,001**

r_s_: Spearman correlation coefficient.

## DISCUSSION

There was no difference between the massage, kangaroo care and control groups in terms of socio-demographic characteristics such as age, education, pregnancy period, number of children, family type, receiving help and planned pregnancy of the mothers (Table 1).

Intra-group comparison of mothers’ maternal function, postpartum anxiety levels, and Beck Depression Scale scores were evaluated in our study (Table 2). The postpartum period is a critical time during which mothers experience significant physical and psychosocial changes and challenges. In cases of infants with colic, inconsolable crying can negatively impact the mother’s mental health and maternal functioning^(35,36,37)^. Research highlights the importance of providing holistic and supportive interventions to mothers of infants with colic in order to improve maternal mental health and functioning^(38,39)^. Upon reviewing the literature, it is found that, similar to our study, mothers who perform infant massage are more sensitive to their infants’ care, behaviors, and development, and they adapt better to the maternal role^(38,39)^. Based on these findings, early nursing education and interventions for mothers of infants with colic are crucial for helping them adapt to and sustain their maternal role. A previous study determined that intermittent kangaroo care significantly increased maternal attachment in mothers of premature infants^(40)^. On the other hand, contrary to our study findings, Lee and Shin^(41)^ determined that kangaroo care had no significant effect on maternal function^(41)^. It is considered that this difference in the findings may be due to the difference in the measurement tools used, the characteristics of the sample groups, or the age of the infants. In our study, an increase in maternal functions was also observed in the control group. This increase may be due to the spontaneous recovery of colic attacks over time, maternal attachment, maternal experiences, and maternal role adaptation. However, the fact that this increase in the control group was limited compared to the massage and kangaroo care groups emphasizes the importance of structured interventions.

The postpartum-specific anxiety posttest scores of the mothers in the massage, kangaroo care, and control groups were found to be significantly lower than the pretest (Table 2). The literature shows that mothers who massage their infants have lower anxiety, anger and burnout levels^(41,42,43,44)^. In the literature, it has been stated that skin-to-skin contact (kangaroo care) reduces the rate of postpartum anxiety, maternal anxiety and sleep problems^(45,46,47)^. In our study, the anxiety level of the mothers in the control group decreased in the post-test. This decrease in the control group can be attributed to the decrease in colic symptoms in infants over time and the increase in mothers’ coping skills by adapting to the process. In conclusion, the implementation of regularly and consciously planned interventions during the postpartum period is a significant clinical and psychosocial necessity for reducing anxiety in mothers of infants with colic. In our study, it was observed that the posttest scores of the depression scale of the mothers in the three groups decreased compared to the pre-test scores (Table 2). This finding is similar to the findings of studies in the literature. Infant massage performed by mothers on healthy infants, premature infants, and low birth weight infants has been shown to significantly reduce anxiety and depression scores of mothers in the postpartum period and improve mood in the near term and one year later^(37,48,49,50,51)^. Similar to our study, kangaroo care has been found in the literature to significantly reduce mothers’ depression, stress, and cortisol levels^(51,52,53,54,55)^. In our study, a decrease in depression scores was also observed in the mothers in the control group. This decrease is thought to be due to the easing of colic symptoms over time, the mother’s better understanding of her baby, and the mother’s experiences. Study findings suggest that kangaroo care helps reduce depression in mothers of colic infants. Therefore, teaching supportive and guiding practices to mothers of infants with infantile colic is crucial to improving their coping skills and mental resilience.

In our study, the increase in maternal function scores for mothers in the massage and kangaroo care group was greater than the increase in scores for mothers in the control group. However, no difference was found between the increase in scores for mothers who received massage and kangaroo care (Table 3). Studies have found that massage and kangaroo care applied to infants are more effective than routine care in improving mothering skills, adaptation to the maternal role, and increasing perceptions of competence^(39,51,52,53,54,55,56)^. In line with these findings, it can be said that planned behavioral nursing interventions positively affect the skills of perceiving and maintaining the maternal role and are more effective than routine care. When the postpartum anxiety levels of the mothers who participated in our study were examined, it was determined that the amount of decrease in the score of the mothers in the control group was less than the massage and kangaroo care groups (Table 3). No difference was found between the massage and kangaroo care groups. When we look at the literature, we found that, similar to our study, the postpartum anxiety levels of mothers who massaged their babies and applied kangaroo care decreased more than those of mothers who applied routine care^(56,57,58,59)^. According to these results, it can be said that both practices have a similar effect in reducing postpartum anxiety in mothers and are more effective than routine care in reducing anxiety. When the amount of decrease in depression scores was analyzed, the highest decrease was found in the mothers in the massage group (Table 3). The amount of decline in the depression score of the mothers in the kangaroo care group was higher than in the control group. According to these findings, it is seen that massage application has the strongest effect on the mental well-being of mothers. Similar to our findings, a study found that massage for babies with colic was more effective in reducing mothers’ postpartum depression compared to other behavioral methods^(51)^. However, although the effectiveness of various interventions for mothers of colicky infants has been measured, little research has compared massage and kangaroo care with alternative methods in their effects on levels of maternal function, postpartum anxiety, and postpartum depression, and has clarified which is superior.

The relationship between infantile colic scores in infants and postpartum anxiety, depression, and maternal functioning in mothers was compared (Table 4). Based on the findings of this study, it can be said that an increase in infantile colic symptoms negatively affects maternal functioning and leads to an increase in anxiety and depression levels. Similar to our study, it has been found in the literature that as colic symptoms increase, insecure attachment, feelings of inadequacy, negative caregiving skills, maternal anxiety, and depression in mothers increase(12,55,60,61).

Our study demonstrated that colic massage and kangaroo care are more effective than routine care in improving postpartum maternal skills, anxiety, and depression. Furthermore, colic massage was found to be more effective in enhancing the maternal role and psychological well-being of mothers with colicky babies. This study is one of the first to directly compare colic massage and kangaroo care in mothers, significant providing evidence on postpartum problems.

## LIMITATIONS OF THE STUDY

The study was conducted in a single hospital with a pediatric outpatient clinic. Furthermore, because the study was conducted in the home setting, mothers’ decisions to participate were based entirely on their own statements. We recommend that future studies be conducted in multiple hospitals with larger sample sizes. We also recommend that similar studies be conducted in healthcare facilities under the supervision of healthcare professionals. No significant difference was found between the groups in terms of infant feeding style. Because no significant differences were found between infant feeding style (breast milk, formula, breast milk, and formula) and infantile colic scale scores, no comparative analysis was conducted on feeding style, maternal function, postpartum anxiety, or postpartum depression. Furthermore, because six mothers withdrew from the study during the study period, an intention-to-treat (ITT) analysis was not performed. Because missing data could not be completed, only data from participants who completed the study were analyzed. This was considered a limitation of our study.

## CONCLUSION AND IMPLICATIONS FOR PRACTICE

This study found that colic massage and kangaroo care reduce maternal anxiety and depression in the postpartum period and enhance maternal function. These findings can serve as a guide for healthcare professionals providing care to mothers during pregnancy and the postpartum period. The application methods of colic massage and kangaroo care can be included in in-service training programs for healthcare professionals, incorporated into prenatal and postnatal education programs, and integrated into public health education campaigns. The long-term outcomes of these practices can be monitored and added to evidence-based guidelines. The study is registered at clinicaltrials.gov NCT06727760.

## Data Availability

The data that support the findings of this study are available from the authors.
