# Supplementary material for: The effect of infantile anti-colic massage and kangaroo care on postpartum anxiety, postpartum depression and maternal function of mothers
Source: Rev Esc Enferm USP. 2026 Jan 23;60:e20250146. doi: 10.1590/1980-220X-REEUSP-2025-0146en (PMC12893339; doi:10.1590/1980-220X-REEUSP-2025-0146en)
Supplement: Translation

# Translation: El efecto del masaje anticólicos infantil y el método canguro en la ansiedad posparto, la depresión posparto y la función materna de las madres*

**DOI:** 10.1590/1980-220X-REEUSP-2025-0146es

**Authors:** Gülçah Sunay Ertem, Nurcan Özyazıcıoğlu

**Affiliations:** 1 Balıkesir State Hospital, Balıkesir, Türkiye.; 2 Bursa Uludağ University, Faculty of Health Sciences, Department of Pediatric Nursing, Bursa, Türkiye.

**Keywords:** Masaje, Método Madre-Canguro, Ansiedad, Depresión Posparto

## Abstract

**Objetivo:**

El objetivo de este estudio fue determinar los efectos del masaje para el cólico posparto y el método canguro sobre la ansiedad, la familia y la productividad materna en las madres.

**Método:**

Este estudio es un ensayo controlado aleatorio. El estudio se llevó a cabo con madres de bebés diagnosticados con cólico infantil. Los grupos del estudio se dividieron aleatoriamente en tres grupos, a saber, masaje, método canguro y grupo de control, mediante aleatorización estratificada. Los datos del estudio se recopilaron utilizando el «Formulario de información sobre la madre y el bebé», el «Índice Barkin de funcionamiento materno (BIMF)», la «Escala de ansiedad específica posparto» y el «Inventario de depresión de Beck (BDI)». La recopilación de datos se completó entre marzo de 2022 y febrero de 2023. El estudio se completó con un total de 90 madres.

**Resultados:**

Los resultados indicaron que el funcionamiento materno mostró la mayor mejora en el grupo de masajes (12,31 ± 5,93, p < 0,001). La disminución de las puntuaciones de ansiedad y depresión posparto fue menor en el grupo de control en comparación con los grupos de masajes y cuidado canguro (5,29 ± 9,51, 1,39 ± 1,28). Se observó una correlación negativa significativa entre el cólico infantil y el funcionamiento materno (rs = -0,69, p < 0,001), mientras que se encontraron correlaciones positivas significativas entre el cólico infantil y la ansiedad y la depresión posparto (rs = 0,51, rs = 0,58, p < 0,001).

**Conclusión:**

El masaje para el cólico y el método canguro deben integrarse en la educación prenatal y posparto rutinaria y deben ser aplicados por las enfermeras en la atención clínica.

## INTRODUCCIÓN

El cólico infantil se define como un llanto excesivo que dura más de tres horas al día, al menos tres días a la semana, y que persiste durante más de tres semanas en los primeros tres meses de vida. Esta afección fue descrita por primera vez por Wessel y sus colegas en 1954^(1)^. El cólico infantil es ampliamente reconocido como una experiencia muy angustiosa para los padres y se asocia particularmente con desafíos psicológicos y familiares entre las madres primerizas^(2)^.

Los estudios han demostrado que los episodios de llanto prolongados e intensos en los bebés con cólico provocan sentimientos de insuficiencia e impotencia en los padres, contribuyen a la depresión posparto y aumentan la necesidad de apoyo social^(3,4)^. Se ha informado de que las madres de bebés con cólico experimentan niveles más altos de depresión, apego inseguro y ansiedad posparto en comparación con las madres de bebés sin cólico^(5,6)^. El estrés y la depresión posparto maternos hacen que las madres cuestionen su competencia para cuidar a sus hijos, lo que dificulta que puedan proporcionarles un cuidado seguro y adecuado^(6)^. Además, se ha informado de que el aumento del estrés parental exacerba los síntomas del cólico infantil^(7)^. Otro estudio identificó una asociación significativa entre los altos niveles de estrés materno y una mayor gravedad de los síntomas del cólico^(8)^. Por otra parte, las investigaciones han demostrado que los problemas psicológicos de la madre durante el periodo prenatal contribuyen a los comportamientos de llanto e irritabilidad de los bebés^(9,10)^.

Una revisión sistemática que examinaba las dificultades de la crianza temprana hizo hincapié en la interdependencia del bienestar del niño y de los padres: las intervenciones que mejoran a los padres también tienen efectos positivos en los niños, mientras que las intervenciones que benefician a los niños favorecen la adaptación psicosocial de los padres^(11)^. Este hallazgo subraya que la interacción entre padres e hijos es un proceso bidireccional y representa un ciclo recíproco de mejora dentro de la dinámica familiar. En el periodo posparto, se ha demostrado que los programas de apoyo a los padres, las intervenciones psicoeducativas, los enfoques de atención centrados en el bebé y los servicios de salud centrados en la familia reducen los niveles de estrés materno y mejoran el bienestar emocional^(11,12,13)^. Se ha descubierto que el masaje infantil y el método canguro disminuyen los niveles de estrés y ansiedad maternos y reducen los marcadores fisiológicos de estrés^(14)^. Los estudios también han demostrado que estas prácticas fortalecen el vínculo entre la madre y el bebé, favorecen la liberación de oxitocina, facilitan la aceptación temprana del papel materno y mejoran la autoeficacia y la sensibilidad maternas en el cuidado^(15,16,17)^.

A pesar de estos prometedores hallazgos, las intervenciones de enfermería diseñadas específicamente para apoyar el bienestar materno y la adaptación al rol de las madres de bebés con cólicos siguen siendo limitadas. Además, la mayoría de los estudios existentes se han centrado predominantemente en intervenciones descritas como “masaje infantil”, lo que deja lagunas en la comprensión de la eficacia comparativa de los diferentes enfoques.

En conclusión, se necesitan nuevos estudios de alta calidad para desarrollar estrategias de afrontamiento para el manejo de los cólicos, apoyar el bienestar materno, determinar qué intervenciones son más eficaces y abordar las lagunas en la literatura. En consonancia con esto, el presente estudio se llevó a cabo para determinar los efectos de dos intervenciones, el “masaje para los cólicos” y el “cuidado canguro”, sobre la depresión posparto, la ansiedad posparto y el funcionamiento materno.

## MÉTODO

### Diseño del Estudio

Este estudio es un ensayo controlado aleatorio, experimental, pretest-postest, realizado para determinar los efectos del masaje anticólicos y el método canguro en el funcionamiento materno, la ansiedad posparto y los niveles de depresión posparto en madres de bebés diagnosticados con cólicos según los criterios de Roma IV.

### Lugar y Momento del Estudio

Los datos para el estudio se recolectaron en la clínica ambulatoria del Departamento de Pediatría del Hospital Municipal Atatürk en dos días seleccionados al azar por semana. Los datos se recolectaron de madres de bebés llevados a la clínica ambulatoria para su examen y diagnosticados con cólico infantil por primera vez por un pediatra. La recolección de datos se completó entre marzo de 2022 y febrero de 2023.

### Población de Investigación y Muestra

Los bebés con cólico infantil recién diagnosticado y sus madres que cumplían los criterios de inclusión fueron incluidos en el estudio entre marzo de 2022 y abril de 2023.

### Tamaño de la Muestra

El tamaño de la muestra del estudio se determinó utilizando G*Power 3.1.9.7. El tamaño del efecto se calculó tanto para los bebés como para las madres utilizando el enfoque ANOVA de medidas repetidas basado en estudios de la literatura. Dado que se observó que el tamaño del efecto de los estudios sobre las madres era menor, se utilizaron cálculos basados en las madres para garantizar la exhaustividad. En consecuencia, con un tamaño del efecto de f = 0,340, una potencia de 0,80 (probabilidad de error 1-β) y α = 0,05 (probabilidad de error de tipo I), se calculó que el tamaño mínimo de la muestra necesario para un diseño con tres grupos y dos mediciones repetidas era de 28 participantes por grupo, lo que daba un total de 84 participantes^(5,18,19)^. Dado que podría haber pérdidas durante el estudio, el tamaño de la muestra se aumentó en un 15%, hasta 96. Se determinó que el tamaño de los grupos fuera de 32 madres en cada uno (masaje, método canguro y grupo de control). Durante el proceso del estudio se llegó a un total de 102 bebés y madres.

Seis parejas de madres e hijos no se incluyeron en el estudio porque no cumplían los criterios de exclusión. Durante el estudio, seis madres abandonaron por diversas razones. El estudio se completó con 90 madres (Figura 1).

**Figura 1:** Flujograma CONSORT.

### Criterios de Inclusión

El estudio incluyó a bebés de sexo masculino y femenino nacidos a término, con un peso entre 2500 y 4500 gramos, diagnosticados con cólico infantil según los criterios de Roma IV, y a sus madres. También se incluyó a las madres que se ofrecieron voluntarias para participar en el estudio, sabían leer y escribir en turco y podían utilizar un teléfono inteligente.

### Criterios de Exclusión

Se excluyó del estudio a los bebés con anomalías congénitas, intolerancia a la lactosa, enfermedades agudas o crónicas, problemas cutáneos, vómitos o diarrea, peso al nacer inferior a 2500 gramos, que recibían tratamiento para el cólico o que desarrollaron alguna enfermedad durante el periodo de estudio. Además, se excluyó del estudio y se consideró perdidas a las madres que no aplicaron al menos una de las intervenciones rutinarias, que aplicaron la intervención de forma incorrecta o que no estaban dispuestas a continuar con el estudio.

### Randomización y Asignación

Los estudios publicados indican que la edad (semanas) y el sexo son factores pronósticos que afectan a los síntomas del cólico infantil^(12,18,19)^. Por lo tanto, se utilizó la aleatorización estratificada para asignar a los bebés y a sus madres a los grupos de intervención y control.

Para controlar los efectos de la edad y el sexo en el dolor del cólico y aumentar la fiabilidad de los resultados, se estratificó a los bebés por edad (3–8 semanas) y sexo (masculino-femenino). La aleatorización de los bebés se realizó introduciendo aleatoriamente todos los números del 1 al 96 a través de https://www.randomizer.org. El primer número extraído se asignó al grupo de masajes, el segundo al grupo de cuidados canguro y el tercero al grupo de control.

### Mediciones

Los datos del estudio se recolectaron utilizando el “Formulario de información sobre la madre y el bebé”, el “Índice Barkin de funcionamiento materno (BIMF)”, la “Escala de ansiedad específica posparto” y el “Inventario de depresión de Beck (BDI)”.

***Formulario de información sobre la madre y el bebé:*** Este formulario, elaborado por el investigador como resultado de una revisión bibliográfica, se finalizó en colaboración con un miembro del cuerpo docente en el campo de la enfermería pediátrica^(2,3,4,5,9,10)^. ”El formulario incluía variables como la edad de las madres, su nivel educativo, su situación laboral y sus ingresos, el número de hijos, el tipo de familia, el consumo de tabaco y alcohol, el consumo de leche de vaca, el método de calefacción del hogar, la presencia de alergias y si recibían apoyo para el cuidado del bebé. También incluía información sobre las visitas al hospital durante los ataques de cólicos”^(20)^. “Además, el formulario incluía variables relacionadas con los bebés, como la edad, el sexo, el tipo de parto, las medidas antropométricas al nacer y en el momento actual, el orden de nacimiento, el método de alimentación, el consumo de biberones y chupetes, la frecuencia diaria de alimentación, la frecuencia diaria de defecación y la hora de inicio y las características de los episodios de llanto”^(20)^.

***El Índice Barkin de Funcionamiento Materno (BIMF):*** Esta escala se desarrolló en 2010 para determinar el estado funcional de la madre después del parto (18). El coeficiente alfa de Cronbach original de la escala es de 0,87 (18). Como resultado de entrevistas grupales con 31 mujeres, se preparó una escala de 20 preguntas, se realizaron estudios de validez y fiabilidad y se publicaron (18). El estudio de validez y fiabilidad de la versión turca del BAFÖ fue completado por Aydın y Kukulu en 2018 (19). Dado que los ítems 15, 16, 18 y 20 del formulario no se consideraron apropiados, se creó una escala turca de 7 puntos tipo Likert, con 16 preguntas y 5 factores, y se determinó que el coeficiente de fiabilidad era de 0,73. Los coeficientes alfa de Cronbach de las subdimensiones de la escala son los siguientes: autocuidado 0,66, psicología materna 0,71, cuidado del bebé 0,62, apoyo social 0,69, y la dimensión de adaptación a la maternidad se calculó en 0,50. Las respuestas de la escala se numera La puntuación más baja que se puede obtener en la escala es 0, y la más alta es 96. Se acepta que el estado funcional aumenta a medida que aumentan las puntuaciones. El índice BAFÖ se aplica a madres con bebés menores de 18 meses^(19)^. Se encontró que el valor del coeficiente alfa de Cronbach del BAFÖ era de 0,87^(19)^.

***Inventario de depresión de Beck (BDI):*** La escala de depresión de Beck se desarrolló en 1961 para medir los hallazgos conductuales de la depresión en adolescentes y adultos^(21)^. El coeficiente alfa de Cronbach original de la escala es de 0,86^(21)^. La validez y fiabilidad de la escala fue realizada por Hisli en 1989 y se encontró que el valor alfa de Cronbach era de 0,80^(22)^. La escala consta de 21 ítems relacionados con síntomas depresivos como pesimismo, sensación de fracaso, insatisfacción, sentimientos de culpa, inquietud, fatiga, disminución del apetito, indecisión, trastornos del sueño y aislamiento social. La puntuación total que se puede obtener de la escala varía entre 0 y 63. En la versión original de la escala, la depresión clínica límite se determinaba entre 17 y 20 puntos. En cuanto a la validez y fiabilidad de la escala en la sociedad turca, se aceptó una puntuación de 17 o más como nivel de “depresión clínicamente significativa”. Estudios de validez y fiabilidad del BDI en turco: la gravedad de la depresión se interpreta como una puntuación del BDI de 0–9 = mínima, 10–16 = leve, 17–29 = moderada, 30–63 = grave^(22)^.

***Escala de ansiedad específica posparto:*** La Escala de ansiedad específica posparto es una escala de 51 ítems desarrollada por Fallon y colegas para evaluar la ansiedad^(23)^. El coeficiente alfa de Cronbach original de la escala es de 0,95, y las subescalas oscilan entre 0,80 y 0,91^(23)^. En el estudio turco sobre la validez y fiabilidad de la Escala de ansiedad específica posparto, se encontró que el coeficiente alfa de Cronbach era de 0,94 (24). Las respuestas a los ítems se califican en una escala Likert de 4 puntos que va de 1 a 4 puntos (1 = nunca, 2 = a veces, 3 = normalmente, 4 = siempre). Puntuación total del nivel de ansiedad posparto: por debajo de 73 puntos se considera riesgo bajo, entre 74 y 100 puntos se considera riesgo medio y 101 puntos o más se considera riesgo alto. La puntuación más baja posible es 51 y la más alta es 204. La escala tiene cuatro subdimensiones: los ítems 1–15 evalúan la adecuación materna y las preocupaciones por el apego, los ítems 16–26 evalúan la seguridad y el bienestar del bebé, los ítems 27–33 evalúan las preocupaciones prácticas sobre el cuidado del bebé y los ítems 34–51 evalúan la adaptación psicosocial a la maternidad^(24)^.

## MATERIALES EDUCATIVOS UTILIZADOS EN EL ESTUDIO

### Lista de Verificación para la Aplicación del Masaje Educativo

La “Lista de verificación para la aplicación del masaje educativo” fue creada por el investigador basándose en la bibliografía y la opinión de un miembro del cuerpo docente del Departamento de Enfermería de Salud y Enfermedades Infantiles. Este material educativo, dividido en tres partes, proporcionaba información sobre los beneficios del masaje infantil, la preparación previa al masaje, la duración del masaje y las situaciones en las que no se debe aplicar el masaje^(25,26,27)^. También se complementaba con imágenes que mostraban las etapas detalladas del masaje para el cólico. Además, se añadió un cuadro con preguntas de sí o no para que la madre pudiera seguir el orden del masaje mientras lo aplicaba.

### Lista de Verificación Educativa para la Aplicación del Método Canguro

La “*Lista de verificación educativa para la aplicación del método canguro”* fue elaborada por el investigador basándose en la bibliografía y la opinión experta de un miembro del cuerpo docente del Departamento de Enfermería de Cuidados de Salud y Enfermedades Infantiles^(28)^. El material educativo constaba de tres secciones e incluía información sobre los beneficios del método canguro, quién podía aplicarlo, cuándo y durante cuánto tiempo debía aplicarse, la posición adecuada del bebé y el entorno apropiado para el método canguro. También contenía imágenes que ilustraban las etapas de la aplicación del método canguro. Además, el material incluía una tabla con preguntas de sí/no para ayudar a las madres a seguir el orden y las reglas correctas de la práctica del método canguro.

### Bebé Modelo

Se utilizó un maniquí de bebé para demostrar el masaje para el cólico y el método canguro.

### Aceite Para Masajes

En las clases de masaje se utilizó aceite hipoalergénico para bebés a fin de lubricar a los bebés.

### Termómetro

El termómetro se utilizó para garantizar que el entorno estuviera a una temperatura adecuada durante el masaje y el método canguro.

## RECOPILACIÓN DE DATOS E INTERVENCIONES

Participaron en el estudio bebés diagnosticados con cólico infantil por un pediatra según los criterios de Roma IV y sus madres. Los criterios diagnósticos de Roma IV son los siguientes:

1. Los síntomas deben desaparecer en los primeros cinco meses desde su aparición.
2. Los episodios de llanto recurrentes y prolongados deben producirse sin una causa aparente y no pueden ser detenidos por los cuidadores.
3. El bebé no debe presentar ningún signo de enfermedad ni retrasos en el crecimiento o el desarrollo^(29)^.

### Intervención del Grupo de Control

Se invitó a participar en el estudio a las madres cuyos bebés habían sido diagnosticados con cólico infantil, y las madres voluntarias rellenaron el “Formulario de consentimiento informado” y el “Formulario de información sobre la madre y el bebé”. A continuación, se administró a las madres la “Escala de función materna de Barkin (BIMF)”, la “Escala de ansiedad específica posparto”, el “Inventario de depresión de Beck” y la “Escala de cólico infantil”. Tres semanas después de las primeras pruebas, se administraron las mismas pruebas a las madres del grupo de control.

### Intervenciones del Grupo de Masajes y del Grupo de Cuidados Canguro

Se recolectaron datos de madres voluntarias de bebés diagnosticados con cólico infantil que cumplían los criterios de inclusión del estudio. Se informó a las madres sobre el estudio mediante el “Formulario de consentimiento informado” y los datos se recolectaron a través de entrevistas presenciales. Antes de las intervenciones, las madres de ambos grupos completaron la “Escala de Funcionamiento Materno de Barkin (BIMF)”, el “Inventario de Depresión de Beck”, la “Escala de Ansiedad Específica Posparto” y la “Escala de Cólico Infantil”. Una vez completadas las pruebas previas y antes de que comenzara la fase de intervención, se programaron dos citas de visitas domiciliarias con las madres del grupo de intervención, con una semana de diferencia. Se realizaron dos visitas domiciliarias en total. Durante la segunda visita, a las madres del grupo de masajes se les enseñó a realizar masajes para el cólico, mientras que a las madres del grupo de cuidado canguro se les enseñó el cuidado canguro. Además, las madres del grupo de intervención continuaron proporcionando cuidados rutinarios a sus bebés durante todo el periodo en el que realizaron los masajes para el cólico. La duración de la intervención se determinó basándose en la bibliografía y en los periodos de implementación utilizados en estudios similares al nuestro^(30,31,32,33)^.

### La Intervención Aplicada al Grupo de Masaje

La enfermera investigadora proporcionó información completa, presencial e individual a las madres del grupo de masaje sobre el masaje para el cólico durante las visitas domiciliarias. A continuación, la enfermera investigadora demostró cómo realizar el masaje para el cólico en un maniquí de bebé. A continuación, las madres realizaron la técnica de masaje para el cólico previamente demostrada en el maniquí de bebé bajo la supervisión de la enfermera investigadora. Las madres repitieron el masaje hasta que fueron capaces de realizarlo correctamente. Esta formación y demostración por parte de la investigadora se impartió a las madres en una sola sesión antes de que comenzara el estudio (la investigadora poseía un certificado de masaje holístico para bebés). Tras la formación y la instrucción, la enfermera investigadora también proporcionó a las madres la “Lista de verificación para la educación sobre el masaje para el cólico”. Tras esta información y demostración, las madres comenzaron a realizar de forma independiente el masaje para el cólico a sus bebés en casa. Durante tres semanas, las madres realizaron un masaje para el cólico en dos fases a sus bebés según la “Lista de verificación para la educación sobre el masaje para el cólico”. Durante tres semanas, realizaron el masaje para el cólico durante 15–20 minutos por la mañana y por la noche, cuando sus bebés estaban tranquilos. Además, cuando los bebés comenzaban a mostrar signos de malestar, las madres realizaban la segunda fase del masaje para el cólico durante 15 minutos^(34)^. El masaje para el cólico (colic massage – CM) constaba de dos partes: el masaje “Te quiero” (I love you o ILU), que se aplicaba cuando el bebé estaba tranquilo, y otra técnica de masaje que se utilizaba cuando el bebé estaba inquieto. Estas técnicas incluían:

1. Cuando el bebé estaba tranquilo, el masaje se aplicaba en el abdomen mientras el bebé estaba acostado en posición supina. Se trazaban suavemente las letras “I”, “L” invertida y “U” invertida en el abdomen del bebé en secuencia. Mientras se mantenía la misma posición, se doblaban las rodillas del bebé. Esta secuencia de masaje se repetía varias veces.
2. Cuando el bebé estaba inquieto, el masaje se realizaba con el bebé mirando hacia adelante en el regazo de la madre. La madre pasaba su brazo izquierdo por debajo de la axila del bebé para sostener el pecho y, con la mano derecha, trazaba suavemente una forma de “U” invertida en el abdomen del bebé en el sentido de las agujas del reloj. A continuación, acariciaba suavemente el abdomen del bebé de arriba abajo con la mano derecha. Por último, mientras el bebé permanecía en el regazo de la madre, esta le doblaba las rodillas y lo balanceaba suavemente de lado a lado^(25,26,27,28).^

### La Intervención Aplicada al Grupo de Cuidado Canguro

Durante las visitas domiciliarias, la enfermera investigadora proporcionó información completa, personalizada y cara a cara sobre el cuidado canguro a las madres del grupo de masajes. A continuación, la enfermera investigadora demostró cómo realizar el cuidado canguro en un bebé de juguete. Las madres, bajo la supervisión de la enfermera investigadora, realizaron el procedimiento de cuidado canguro con el bebé de juguete. Las madres repitieron el procedimiento de cuidado canguro hasta que fueron capaces de realizarlo correctamente. Esta formación y demostración, proporcionada por la investigadora, se impartió a las madres en una sola sesión antes de que comenzara el estudio. Tras la formación y la demostración, la enfermera investigadora entregó a las madres la “Lista de verificación para la educación sobre el cuidado canguro”. Tras esta visita domiciliaria de la enfermera investigadora, las madres comenzaron a aplicar de forma independiente el método canguro a sus bebés en casa. Aplicaron el método canguro durante 15–20 minutos por la mañana y por la noche, cuando el bebé estaba tranquilo, y durante 15 minutos adicionales cuando el bebé comenzaba a mostrar signos de malestar^(17,33)^.

Una vez finalizadas las intervenciones, las madres de ambos grupos rellenaron la “Escala de Funcionamiento Materno de Barkin (BIMF)”, el “Inventario de Depresión de Beck”, la “Escala de Ansiedad Específica Posparto” y la “Escala de Cólicos Infantiles”. A lo largo de la fase de intervención, las madres recibieron apoyo y orientación a través de videollamadas según fuera necesario. Cuando las videollamadas no eran factibles, se pidió a las participantes que enviaran grabaciones de vídeo de sus sesiones de práctica. (La enfermera investigadora realizó la aplicación a modo de ejemplo en los videos solicitados a las madres y en las entrevistas en video que mantuvo con ellas. La enfermera investigadora no participó en la aplicación que las madres debían realizar en el marco de la investigación. Las madres realizaron por sí mismas las aplicaciones matutinas y vespertinas especificadas en las normas de aplicación. Se contactó a las madres a través de WhatsApp para recordarles las intervenciones. Esto garantizó que las intervenciones se llevaran a cabo dentro del tiempo especificado. El estudio duró un total de cuatro semanas, y la fase de intervención ocupó las tres primeras semanas.

### Análisis de Datos

La introducción de datos en el programa estadístico se realizó asignando códigos a los grupos. Los grupos de intervención y control se codificaron como 1, 2 y 3. La persona que realizó el análisis de datos desconocía la asignación de los grupos. Se utilizó la prueba de Shapiro-Wilk para evaluar la normalidad de las variables continuas. Las variables continuas se presentaron como valores medios, desviaciones estándar o medianas (mínimo: máximo). Las variables categóricas se expresaron como n (%). Si los datos se distribuían normalmente o si había dos o más grupos, se utilizó la prueba ANOVA; en caso contrario, se aplicó la prueba de Kruskal-Wallis. Si se encontraba una significación global en el análisis avanzado de subgrupos, a la prueba ANOVA le seguía la prueba de Bonferroni, y a la prueba de Kruskal-Wallis le seguía la prueba de Dunn-Bonferroni. (Los análisis estadísticos avanzados de nuestro estudio se presentan en la Tabla 4). En la comparación de los resultados previos y posteriores a la prueba, si se observaba normalidad, se utilizó la prueba t para muestras emparejadas; en caso contrario, se aplicó la prueba de rangos con signo de Wilcoxon. Además, se utilizaron las pruebas de chi cuadrado y Fisher Freeman Halton para la comparación de variables categóricas entre grupos. Los análisis estadísticos se realizaron con el software SPSS (IBM Corp. Lanzado en 2012. IBM SPSS Statistics para Windows, versión 25,0. Armonk, NY: IBM Corp.), y el nivel de error de tipo I se fijó en el 5%. Además, las comparaciones longitudinales fundamentales del manuscrito se basaron en las puntuaciones de cambio previas y posteriores a la intervención, evaluadas con pruebas adecuadas dentro del grupo y entre grupos, elegidas en función de las propiedades de distribución de los datos (paramétricas cuando se cumplían los supuestos y no paramétricas en caso contrario). No realizamos un ANOVA clásico de medidas repetidas para estimar la interacción Grupo × Tiempo. No planeamos introducir modelos de efectos mixtos post hoc u otras pruebas de interacción “avanzadas”, porque se infringieron los supuestos estadísticos clave necesarios para esos modelos en varios resultados primarios. Concretamente, las diferentes distribuciones mostraban desviaciones sustanciales de la normalidad, se observaba heterogeneidad de la varianza entre los grupos y, en el caso de algunas variables, la estructura de los datos (por ejemplo, la asimetría y la presencia de valores atípicos en resultados como la duración del llanto) haría que las estimaciones de interacción de los modelos factoriales paramétricos fueran poco fiables o engañosas. Forzar una prueba de interacción Grupo × Tiempo en estas condiciones, sin una corrección confiable o una muestra sustancialmente mayor para estabilizar las estimaciones, correría el riesgo de generar resultados espurios (error de tipo I inflado por violaciones de esfericidad u homocedasticidad), estimaciones de efectos sesgadas o inestables e inferencias excesivamente confiables (intervalos de confianza reducidos que no reflejan la verdadera incertidumbre). Del mismo modo, la introducción de modelos complejos en esta fase rozaría el sobreajuste y podría oscurecer, en lugar de aclarar, la señal práctica de la intervención. Por lo tanto, consideramos que el enfoque inicialmente descrito, centrado en las puntuaciones de cambio con una selección adecuada de pruebas, proporciona el resumen más transparente y estadísticamente defendible del cambio diferencial a lo largo del tiempo entre los grupos, dados los datos disponibles.

### Cuestiones Éticas

Este estudio se llevó a cabo de conformidad con la Declaración de Helsinki. La aprobación ética del estudio fue otorgada por el Comité de Ética en Investigación No Intervencionista de la Universidad de Ciencias de la Salud de Bandırma On Yedi Eylül (aprobación n.º 72 de fecha 17,12,2021). El permiso institucional para llevar a cabo el estudio en el Hospital Municipal Atatürk fue otorgado por la Dirección Provincial de Salud de Balıkesir (aprobación de la Comisión de fecha 07,03,2022). Se siguieron todas las directrices éticas para la investigación con seres humanos. Se obtuvo el consentimiento por escrito de las madres mediante el “Formulario de consentimiento informado” antes de comenzar la etapa de recolección de datos. Se informó a las participantes de que podían retirarse del estudio en cualquier momento. El estudio está registrado en clinicaltrials. gov NCT06727760.

## RESULTADOS

Los datos recolectados de acuerdo con los objetivos del estudio experimental de diseño pretest-postest para revelar el efecto del masaje anticólicos y el método canguro en los bebés con cólicos se analizaron estadísticamente. Se compararon las características sociodemográficas de las madres entre los grupos. Los datos revelaron que no había diferencias entre los grupos en cuanto a la edad de las madres, la educación, el período de embarazo, el número de hijos, el tipo de familia, la ayuda que recibían las madres y el embarazo planificado (Tabla 1).

**Tabla 1:** Comparación de las características sociodemográficas de las madres entre los grupos – Balıkesir, región de Mármara, Turquía, 2023.

Características	Grupo masaje		Grupo cuidado canguro		Grupo control		p-valor
	n	%	n	%	n	%	
**Nivel de educación materna**							
Primaria	4	13,80	3	10	1	3,20	0,689^b^
Secundaria	9	31	11	36,70	11	35,50	
Curso superior o más	16	55,20	16	53,30	19	61,30	
**Período del embarazo**							
Bueno (saludable)	20	69	17	56,70	13	41,90	0,108^a^
Malo (con problemas de salud)	9	31	13	43,30	18	58,10	
**Tipo familiar**							
Nuclear	20	69	20	66,70	21	67,70	0,919^b^
Extendida	3	10,30	4	13,30	2	6,50	
Extendida temporaria	6	20,70	6	20	8	25,80	
**Estaus de apoyo de la madre**							
Si	22	75,90	24	80	23	74,20	0,859^a^
No	7	24,10	6	20	8	25,80	
**Embarazo planeado**							
Si	17	58,60	17	56,70	16	51,60	0,852^a^
No	12	41,40	13	43,30	15	48,40	
**Número de hijos**	1,79 ± 0,82		1,70 ± 0,65		1,77 ± 0,72		0,937^c^
**Edad de la madre**	28,79 ± 4,99		28,33 ± 4,59		28,97 ± 5,08		0,875^c^

Los datos se expresan como media ± desviación estándar, n%.

a: prueba de chi cuadrado,

b: prueba de Fisher-Freeman-Halton,

c: prueba de Kruskal Wallis.

Se compararon las puntuaciones intragrupales de las madres en el Índice Barkin de Funcionamiento Materno (BIMF), la Escala de Ansiedad Específica Posparto y el Inventario de Depresión de Beck (BDI) (Tabla 2). Las puntuaciones posteriores a la prueba del Índice Barkin de Funcionamiento Materno de las madres de los grupos de masaje, cuidado canguro y control fueron más altas en comparación con sus puntuaciones previas a la prueba (p < 0,001). Las puntuaciones posteriores a la prueba de la Escala de Ansiedad Específica Posparto de las madres de los grupos de masaje, cuidado canguro y control fueron inferiores en comparación con sus puntuaciones previas a la prueba (p < 0,001, Tabla 2). Las puntuaciones posteriores a la prueba del Inventario de Depresión de Beck de las madres de los grupos de masaje, cuidado canguro y control fueron inferiores en comparación con sus puntuaciones previas a la prueba (p < 0,001, Tabla 2).

**Tabla 2:** Comparación intragrupal de los índices de Barkin sobre el funcionamiento materno, la ansiedad específica posparto y las puntuaciones de la escala de depresión de Beck de las madres – Balıkesir, región de Mármara, Turquía, 2023.

	Grupo masaje	Grupo cuidado canguro	Grupo control
Índice Barkin de Funcionamiento Materno (BIMF) Puntuación pre-test	67,62 ± 11,25	74,83 ± 6,18	65,97 ± 6,78
Índice Barkin de Funcionamiento Materno (BIMF) Puntuación post-test	79,93 ± 8,24	82,63 ± 7,42	68,48 ± 7,50
p-valor	**< 0,001^e^**	**< 0,001^e^**	**< 0,001^e^**
Puntuación de ansiedad específica posparto pre-test	100,69 ± 20,96	95,13 ± 15,87	109,03 ± 14,55
Puntuación de ansiedad específica posparto post-test Puntuación	79,48 ± 15,89	82,47 ± 14,88	103,74 ± 10,84
p-valor	**< 0,001^d^**	**< 0,001^e^**	**0,004^e^**
Puntuación del Inventario de Depresión de Beck pre-test	18,14 ± 8,80	12,57 ± 4,97	13,48 ± 3,41
Puntuación del Inventario de Depresión de Beck post-test	10,41 ± 5,17	9,33 ± 4,47	12,09 ± 3,18
p-valor	**< 0,001^e^**	**< 0,001^d^**	**< 0,001^d^**

Los datos se expresan como media ± desviación estándar.

d: prueba de rangos con signo de Wilcoxon,

e: prueba t para muestras dependientes.

Se calculó la diferencia entre las puntuaciones pre-test y post-test de las madres en el Índice Barkin de Funcionamiento Materno, la Escala de Ansiedad Específica Posparto y el Inventario de Depresión de Beck (Tabla 3). Se observó que el aumento en las puntuaciones del Índice Barkin de Funcionamiento Materno de las madres del grupo de control era menor en comparación con las del grupo de masajes y el grupo de cuidado canguro (p < 0,001 y p < 0,001, Tabla 3). Se observó que la disminución en las puntuaciones de la Escala de Ansiedad Específica Posparto de las madres del grupo de control era menor en comparación con las de los grupos de masaje y cuidado canguro (p < 0,001 y p < 0,002, Tabla 3). No se observaron diferencias entre los grupos de masaje y de cuidado canguro en cuanto a las puntuaciones del post-test sobre el funcionamiento materno y la ansiedad posparto (p = 0,090 y p = 0,104, Tabla 3). Se observó que la disminución en las puntuaciones del Inventario de Depresión de Beck de las madres del grupo de masajes era mayor en comparación con las del grupo de control y el grupo de cuidado canguro (p = 0,001 y p < 0,001, Tabla 3). Se observó que la disminución en las puntuaciones del Inventario de Depresión de Beck de las madres del grupo de cuidado canguro era mayor en comparación con el grupo de control (p = 0,013, Tabla 3).

**Tabla 3:** Comparación de las puntuaciones de las madres en las diferencias intergrupales en el Índice de Funcionamiento Materno de Barkin, la Ansiedad Específica Posparto y las Escalas de Depresión de Beck – Balıkesir, región de Mármara, Turquía, 2023.

					Análisis por sub-grupos		
	Grupo masaje	Grupo cuidado canguro	Grupo control	p-valor	Grupo de masajes y cuidado canguro Valor	Grupo de masaje y grupo de control p	Cuidado canguro y grupo de control p-valor
Índice de Barkin de Funcionamiento Materno (BIMF) Puntuación de diferencia	12,31 ± 5,93	7,80 ± 4,70	2,51 ± 2,60	**< 0,001^c^**	0,090	**< 0,001**	**< 0,001**
Puntuación de diferencia de ansiedad específica posparto	21,21 ± 12,97	12,67 ± 8,8	5,29 ± 9,51	**< 0,001^c^**	0,104	**< 0,001**	**0,002**
Puntuación de diferencia de la Escala de Depresión de Beck	-7,72 ± 4,59	3,23 ± 2,88	1,39 ± 1,28	**< 0,001^c^**	**0,001**	**< 0,001**	**0,013**

Los datos se expresan como media ± desviación estándar,

c: prueba de Kruskal Wallis,

f: prueba ANOVA.

Se calculó la relación entre los síntomas de cólico infantil de los bebés y el funcionamiento materno, la ansiedad posparto y el estado de depresión de las madres (Tabla 4). Se encontró una correlación inversa significativa entre la puntuación diferencial de la Escala de Cólico Infantil y la puntuación diferencial de la Escala de Funcionamiento Materno de Barkin (Coeficiente de correlación de Spearman - r_s_) = -0,69, p < 0,001). Se encontró una correlación positiva significativa entre la puntuación diferencial de la Escala de Cólico Infantil y la puntuación diferencial de la Ansiedad Específica Posparto (r_s_ = 0,51, p < 0,001). Se encontró una correlación positiva significativa entre la puntuación diferencial de la Escala de Cólico Infantil y la puntuación diferencial del Inventario de Depresión de Beck (r_s_ = 0,58, p < 0,001).

**Tabla 4:** Correlación entre la puntuación de la escala de cólicos infantiles y las puntuaciones de Barkin sobre la función materna, la ansiedad posparto específica y el inventario de depresión de Beck – Balıkesir, región de Mármara, Turquía, 2023.

	Puntuación de diferencia en la escala de cólico infantil
Indice Barkin de Funcionamiento Materno (BIMF) Puntuación de diferencia	r_s_ = -0,69 p = **< 0,001**
Ansiedad específica posparto Puntuación de diferencia	r_s_ = 0,51 p = **< 0,001**
Escala de Depresión de Beck Puntuación de diferencia	r_s_ = 0,58 p = **< 0,001**

## DISCUSIÓN

No hubo diferencias entre los grupos de masaje, cuidado canguro y control en cuanto a características sociodemográficas como la edad, la educación, el periodo de embarazo, el número de hijos, el tipo de familia, la recepción de ayuda y el embarazo planificado de las madres (Tabla 1).

En nuestro estudio se evaluó la comparación intragrupal de la función materna, los niveles de ansiedad posparto y las puntuaciones en la Escala de Depresión de Beck de las madres (Tabla 2). El periodo posparto es un momento crítico durante el cual las madres experimentan cambios y retos físicos y psicosociales significativos. En los casos de bebés con cólicos, el llanto inconsolable puede afectar negativamente a la salud mental y al funcionamiento materno de la madre^(35,36,37)^. Las investigaciones destacan la importancia de proporcionar intervenciones holísticas y de apoyo a las madres de bebés con cólicos para mejorar la salud mental y la función materna^(38,39)^. Al revisar la literatura, se observa que, al igual que en nuestro estudio, las madres que realizan masajes infantiles son más sensibles al cuidado, el comportamiento y el desarrollo de sus bebés, y se adaptan mejor al papel materno^(38,39)^. Basándonos en estos hallazgos, la educación y las intervenciones tempranas en materia de lactancia para las madres de bebés con cólicos son fundamentales para ayudarlas a adaptarse y mantener su función maternal. Un estudio anterior determinó que el método canguro intermitente aumentaba significativamente el apego maternal en las madres de bebés prematuros^(40)^. Por otro lado, contrariamente a los resultados de nuestro estudio, Lee y Shin^(41)^ determinaron que el método canguro no tenía un efecto significativo en la función materna^(41)^. Se considera que esta diferencia en los resultados puede deberse a la diferencia en las herramientas de medición utilizadas, las características de los grupos de muestra o la edad de los bebés. En nuestro estudio, también se observó un aumento de las funciones maternas en el grupo de control. Este aumento puede deberse a la recuperación espontánea de los ataques de cólico con el tiempo, al apego materno, a las experiencias maternas y a la adaptación al papel materno. Sin embargo, el hecho de que este aumento en el grupo de control fuera limitado en comparación con los grupos de masaje y cuidado canguro subraya la importancia de las intervenciones estructuradas.

Las puntuaciones de la prueba posterior específica para la ansiedad posparto de las madres de los grupos de masaje, método canguro y control fueron significativamente más bajas que las de la prueba previa (Tabla 2). La literatura muestra que las madres que dan masajes a sus bebés tienen niveles más bajos de ansiedad, ira y agotamiento^(41,42,43,44)^. En la literatura se ha afirmado que el contacto piel con piel (método canguro) reduce la tasa de ansiedad posparto, la ansiedad materna y los problemas de sueño^(45,46,47)^. En nuestro estudio, el nivel de ansiedad de las madres del grupo de control disminuyó en el posttest. Esta disminución en el grupo de control puede atribuirse a la disminución de los síntomas de cólico en los bebés con el tiempo y al aumento de la capacidad de afrontamiento de las madres al adaptarse al proceso. En conclusión, la implementación de intervenciones planificadas de forma regular y consciente durante el periodo posparto es una necesidad clínica y psicosocial importante para reducir la ansiedad en las madres de bebés con cólicos. En nuestro estudio, se observó que las puntuaciones del post-test de la escala de depresión de las madres de los tres grupos disminuyeron en comparación con las puntuaciones del pre-test (Tabla 2). Este hallazgo es similar a los resultados de los estudios de la literatura. Se ha demostrado que el masaje infantil realizado por las madres a bebés sanos, prematuros y con bajo peso al nacer reduce significativamente las puntuaciones de ansiedad y depresión de las madres en el posparto y mejora el estado de ánimo a corto plazo y un año después^(37,48,49,50,51)^. Al igual que en nuestro estudio, en la literatura se ha observado que el método canguro reduce significativamente la depresión, el estrés y los niveles de cortisol de las madres^(51,52,53,54,55)^. En nuestro estudio, también se observó una disminución de los niveles de depresión en las madres del grupo de control. Se cree que esta disminución se debe a la disminución de los síntomas del cólico con el tiempo, a una mejor comprensión del bebé por parte de la madre y a las experiencias de esta. Los resultados del estudio sugieren que el método canguro ayuda a reducir la depresión en las madres de bebés con cólico. Por lo tanto, enseñar prácticas de apoyo y orientación a las madres de bebés con cólico infantil es fundamental para mejorar sus habilidades de afrontamiento y su resiliencia mental.

En nuestro estudio, el aumento de las puntuaciones de la función materna de las madres del grupo de masaje y cuidado canguro fue mayor que el aumento de las puntuaciones de las madres del grupo de control. Sin embargo, no se encontraron diferencias entre el aumento de las puntuaciones de las madres que recibieron masaje y las que recibieron cuidado canguro (Tabla 3). Los estudios han demostrado que el masaje y el método canguro aplicados a los bebés son más eficaces que los cuidados rutinarios para mejorar las habilidades maternales, la adaptación al papel de madre y aumentar la percepción de competencia^(39,51,52,53,54,55,56)^. En consonancia con estos hallazgos, se puede afirmar que las intervenciones de enfermería conductual planificadas influyen positivamente en las habilidades de percepción y mantenimiento del papel de madre y son más eficaces que los cuidados rutinarios. Al examinar los niveles de ansiedad posparto de las madres que participaron en nuestro estudio, se determinó que la disminución de la puntuación de las madres del grupo de control era menor que la de los grupos de masaje y método canguro (Tabla 3). No se encontraron diferencias entre los grupos de masaje y método canguro. Al consultar la bibliografía, se observó que, al igual que en nuestro estudio, los niveles de ansiedad posparto de las madres que masajeaban a sus bebés y aplicaban el método canguro disminuyeron más que los de las madres que aplicaban la atención rutinaria^(56,57,58,59)^. Según estos resultados, se puede afirmar que ambas prácticas tienen un efecto similar en la reducción de la ansiedad posparto en las madres y son más eficaces que la atención rutinaria para reducir la ansiedad. Al analizar la disminución de las puntuaciones de depresión, se observó que la mayor disminución se produjo en las madres del grupo de masajes (Tabla 3). La disminución de la puntuación de depresión de las madres del grupo de cuidado canguro fue mayor que la del grupo de control. Según estos resultados, se observa que la aplicación de masajes tiene el efecto más fuerte sobre el bienestar mental de las madres. De manera similar a nuestros hallazgos, un estudio encontró que el masaje para bebés con cólicos era más eficaz para reducir la depresión posparto de las madres en comparación con otros métodos conductuales^(51)^. Sin embargo, aunque se ha medido la eficacia de diversas intervenciones para las madres de bebés con cólicos, pocas investigaciones han comparado el masaje y el método canguro con métodos alternativos en cuanto a sus efectos sobre los niveles de función materna, la ansiedad posparto y la depresión posparto, y han aclarado cuál es superior.

Se comparó la relación entre las puntuaciones de cólicos infantiles en los bebés y la ansiedad posparto, la depresión y la función materna en las madres (Tabla 4). Según los resultados de este estudio, se puede afirmar que un aumento de los síntomas de cólicos infantiles afecta negativamente a la función materna y conduce a un aumento de los niveles de ansiedad y depresión. Al igual que en nuestro estudio, se ha observado en la literatura que, a medida que aumentan los síntomas de cólico, aumentan el apego inseguro, los sentimientos de insuficiencia, las habilidades negativas para el cuidado, la ansiedad materna y la depresión en las madres^(12,55,60,61)^.

Nuestro estudio demostró que el masaje para el cólico y el método canguro son más eficaces que los cuidados rutinarios para mejorar las habilidades maternas posparto, la ansiedad y la depresión. Además, se observó que el masaje para el cólico era más eficaz para mejorar el papel materno y el bienestar psicológico de las madres con bebés con cólicos. Este estudio es uno de los primeros en comparar directamente el masaje para el cólico y el método canguro en las madres, lo que proporciona pruebas significativas sobre los problemas posparto.

## LIMITACIONES DEL ESTUDIO

El estudio se llevó a cabo en un solo hospital con una clínica pediátrica ambulatoria. Además, dado que el estudio se realizó en el entorno doméstico, la decisión de las madres de participar se basó exclusivamente en sus propias declaraciones. Recomendamos que los estudios futuros se realicen en varios hospitales con muestras de mayor tamaño. También recomendamos que se realicen estudios similares en centros de salud bajo la supervisión de profesionales sanitarios. No se encontraron diferencias significativas entre los grupos en cuanto al estilo de alimentación de los bebés. Dado que no se encontraron diferencias significativas entre el estilo de alimentación de los bebés (leche materna, fórmula, leche materna y fórmula) y las puntuaciones de la escala de cólicos infantiles, no se realizó ningún análisis comparativo sobre el estilo de alimentación, la función materna, la ansiedad posparto o la depresión posparto. Además, dado que seis madres se retiraron del estudio durante el periodo de estudio, no se realizó un análisis por intención de tratar (ITT). Dado que no se pudieron completar los datos que faltaban, solo se analizaron los datos de las participantes que completaron el estudio. Esto se consideró una limitación de nuestro estudio.

## CONCLUSIÓN E IMPLICACIONES PARA LA PRÁCTICA

Este estudio descubrió que el masaje para el cólico y el método canguro reducen la ansiedad y la depresión maternas en el posparto y mejoran la función materna. Estos hallazgos pueden servir de guía para los profesionales sanitarios que atienden a las madres durante el embarazo y el posparto. Los métodos de aplicación del masaje para el cólico y el método canguro pueden incluirse en los programas de formación continua para profesionales de la salud, incorporarse a los programas de educación prenatal y posnatal e integrarse en las campañas de educación para la salud pública. Los resultados a largo plazo de estas prácticas pueden monitorearse y añadirse a las directrices basadas en la evidencia.

## Data Availability

Los datos que respaldan los hallazgos de este estudio están disponibles a través de los autores.
